# Advances in Small RNA Regulation of Female Gametophyte Development in Flowering Plants

**DOI:** 10.3390/plants14091286

**Published:** 2025-04-23

**Authors:** Yanfen Liu, Qing He, Han Su, Xinpeng Xi, Xiaoyuan Xu, Yuan Qin, Hanyang Cai

**Affiliations:** 1 College of Life Sciences, Fujian Agriculture and Forestry University, Fuzhou 350002, China; yanfen.liu@fafu.edu.cn (Y.L.); suhan@fafu.edu.cn (H.S.); 18235795238@163.com (X.X.); xuxiaoyuan@fafu.edu.cn (X.X.); 2 Fujian Provincial Key Laboratory of Haixia Applied Plant Systems Biology, Fujian Agriculture and Forestry University, Fuzhou 350002, China; 3 State Key Laboratory of Ecological Pest Control for Fujian and Taiwan Crops, Fujian Agriculture and Forestry University, Fuzhou 350002, China; 4 Haixia Institute of Science and Technology, Fujian Agriculture and Forestry University, Fuzhou 350002, China; 5 Department of Biosciences, Durham University, Durham DH1 3LE, UK; qing.he3@durham.ac.uk

**Keywords:** angiosperms, female gametophyte, megagametogenesis, megasporogenesis, ovule development, *miRNA*, *siRNA*, *ta-siRNA*

## Abstract

Female gametophyte development in flowering plants is a highly intricate process involving a series of tightly regulated biological events, including the establishment and differentiation of a macrospore mother cell (MMC), the formation of a functional macrospore (FM), and the subsequent development of the embryo sac. The seamless progression of these events is crucial for the completion of sexual reproduction and the alternation of generations in plants. Small RNAs are ubiquitously present in eukaryotic organisms. Based on their biogenesis, function, and involvement in biological pathways, plant small RNAs are primarily categorized into four classes: *miRNAs* (*microRNAs*), *ta-siRNAs* (*trans-acting-siRNAs*), *hc-siRNAs* (*heterochromatic-siRNAs*), and *nat-siRNAs* (*natural antisense transcript-derived siRNAs*). Current studies show that small RNAs play an important role in plant reproductive development, such as female gametophyte development and ovule development. In this review, we systematically elucidate the biogenesis and molecular mechanism of small RNAs and summarize the latest research advances on their roles in regulating megasporogenesis and megagametogenesis in plants. The aim of this review is to provide insights into the mechanisms underlying plant reproductive development through the lens of small RNAs, offering a theoretical foundation for improving crop quality, yield, genetic improvement, and breeding.

## 1. Introduction

The life cycle of angiosperms encompasses the complete process from seed germination to the formation of a new seed generation. This cycle consists of two distinct stages: the sporophyte stage and the gametophyte stage. Specifically, the development of male and female gametophytes, the process of double fertilization, and the formation and germination of seeds are three critical aspects that ensure the emergence and continuation of the alternating generations cycle in plants. The development of female gametophytes in flowering plants is a critical prerequisite for plant sexual reproduction. Unlike animals, which form germinal stem cells during early embryonic development, plants undergo a process of redevelopment. In this process, specific somatic cells within the pistil of adult plants give rise to germ cell precursors, which then develop into germ cells [1]. The female gametophyte’s successful development is fundamental for plants to complete double fertilization, produce offspring, and significantly impacts the quality and yield of crops. The female gametophyte’s development in flowering plants is primarily divided into two successive stages: megasporogenesis and megagametogenesis, involving numerous biological processes and complex regulatory events. Megasporogenesis begins with the archesporial cell (AC), a somatic cell in the apical subepithelial layer (L2 layer) of the ovule primordium, which further specializes and expands to form the megaspore mother cell (MMC). The MMC undergoes one round of meiosis to form four haploid megaspores. In the process of development, the three megaspores typically degenerate; nevertheless, only one megaspore adjacent to the chalazal, the functional megaspore (FM), survives [2]. The megagametogenesis stage encompasses the FM, which develops and undergoes three successive mitotic divisions to form a syncytium, cellularization of the syncytium, and specialization of cell fates, ultimately resulting in the mature embryo sac with the characteristic “seven cells and eight nuclei” configuration [2]. The female gametophyte typically comprises four types of cells: egg cells, synergid cells, central cells, and antipodal cells. In some flowering plants, such as *Arabidopsis thaliana*, the female reproductive unit mainly consists of synergids, the egg cell, and the central cell, while the antipodal cells are completely degraded and degenerated prior to fertilization. As development proceeds, the egg cell and the central cell within the ovule are each fertilized by a sperm cell, leading to the formation of the zygote and the triploid endosperm, respectively. This unique double fertilization process is a hallmark of angiosperms, facilitating the completion of sexual reproduction [3,4].

In recent years, the development and maturation of the female gametophyte in flowering plants have emerged as an excellent system for comprehending cell differentiation and genetic regulatory networks. Research into its cytological processes and related molecular mechanisms has risen to prominence within the field of plant biology. Small RNAs, which play a pivotal role in plant reproduction and development, function by either degrading target gene mRNAs or inhibiting their translation process. Since the initial discovery of small RNAs in *Arabidopsis thaliana* by Bartel [5], Carrington [6], and Chen [7] in 2002, these small RNA molecules have rapidly emerged as a central focus in plant molecular biology research. Current research on plant small RNAs predominantly concentrates on higher plants such as *Arabidopsis thaliana* [8,9,10,11], *Oryza sativa* [12,13,14], *Zea mays* L. [15], *Triticum aestivum* L. [16], *Populus* L. [17], *Solanum lycopersicum* L. [18,19], etc. These studies largely explore the different types of small RNAs and their regulatory mechanisms in key biological processes, including root development [20,21], leaf morphogenesis [22], flowering time [23], flower morphogenesis [24], and responses to various environmental stresses [25,26,27,28,29]. However, the role of small RNAs in the development of the female gametophyte, one of the most complex organogenic processes in plants, remains underreported.

In this review, we focus on the model plant, *Arabidopsis thaliana,* to synthesize current advances on the regulatory roles of small RNAs during megasporogenesis and megagametogenesis. Through systematic dissection of small RNA functions during female gametophyte development, we elucidate their mechanistic roles in orchestrating key molecular events underlying plant reproductive processes.

## 2. The Biogenesis, Molecular Mechanisms, and Biological Functions of Small RNAs

Small RNAs, endogenous non-coding single-stranded small RNA molecules approximately 20–25 nucleotides (nt) in length, are prevalent in eukaryotes and account for approximately 2% of the entire genome. Mature small RNAs associate with members within the ARGONAUTE (AGO) family of proteins to form the RNA-induced silencing complex (RISC), which mediates the post-transcriptional repression of gene expression [30]. As research into the biosynthetic pathways, functional mechanisms, and stress responses of small RNAs expands, these molecules have emerged as “star molecules” in the field of biological research. In plants, small RNAs are primarily categorized into four classes based on their biosynthetic pathways, molecular mechanisms, and biological functions: *miRNAs*, *ta-siRNAs*, *hc-siRNAs,* and *nat-siRNAs*.

### 2.1. miRNA Synthesis, Molecular Mechanisms, and Biological Functions

**Figure 1:**
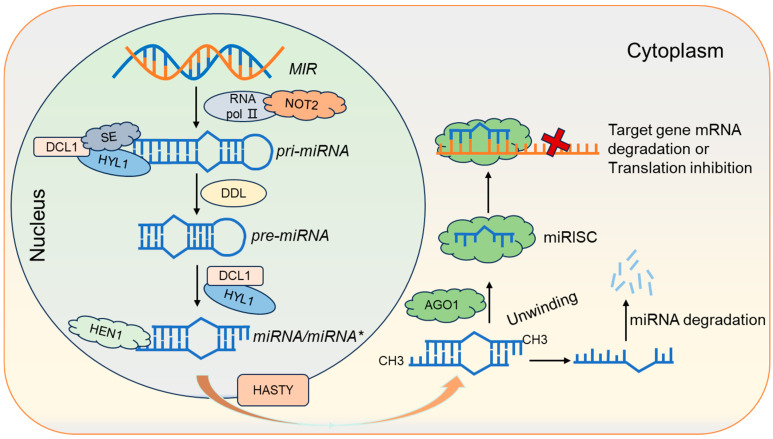
*miRNA* biogenesis and degradation. The *MIR* gene is transcribed into a *pre-miRNA,* followed by processing, including initial generation of a miRNA/miRNA* duplex, methylation modification, nuclear export, and assembly with AGO1 protein complexes, to degrade target gene mRNAs or inhibit translation of target gene mRNAs. In this figure, miRNA/miRNA*: the guide strand (*miRNA*) is loaded into RISC, while the passenger strand (miRNA*) is degraded during assembly. Black arrows indicate activation processes; orange arrows represent nuclear export of miRNA/miRNA*; and red crosses denote target mRNA degradation or translational repression.

*MicroRNA* (*miRNA*) is an endogenous 20–24 nucleotide non-coding RNA that mediates post-transcriptional gene regulation. *miRNA* undergoes a complex and precise series of cleavage and processing steps from initial transcripts to final maturation, occurring within both the cytoplasm and nucleus in plants [31,32]. The process primarily includes the following (Figure 1) [33,34,35]: Firstly, similar to protein-coding genes, *MIR* genes are transcribed in the nucleus under the regulation of a series of factors such as *NOT2*, *RNA polymerase II* (*Pol II*), and other specific transcription factors. This transcription produces *primary miRNA* (*pri-miRNA*) molecules, which are several hundred to several thousand bases in length, possess a 5′ end cap structure and a 3′ poly-A tail, and contain one or more hairpin stem-loop structures [36,37]. Then, the *miRNA* processing complex, comprising DICER-LIKE 1 (DCL1)-SERRATE (SE)-HYPONASTIC LEASES 1 (HYL1), recognizes and binds to specific stem-loop structures of *pri-miRNAs* [38,39,40,41,42]. It also recruits DAWDLE (DDL) proteins for further processing, resulting in the formation of 70 nt *pre-miRNAs* (*precursor miRNAs*) [43]. Subsequently, the DCL1-HYL1 complex processes the *pre-miRNAs* into a mature *miRNA* double-stranded complex (*miRNA*/miRNA*) [36,44].

To ensure stability and prevent degradation, the methyltransferase HUA ENHANCER 1 (HEN1) modifies the *miRNA* complex via methylation, thereby completing the nuclear phase of *miRNA* transcription and processing [45,46]. Following methylation modification, the double-stranded *miRNA*/miRNA* molecules are transported from the nucleus to the cytoplasm for further modification and processing, facilitated by the HASTY (HST) protein [47]. Ultimately, the double helix unwinds, with one strand being rapidly degraded [48], while the other strand binds to the RNA-induced silencing complex (RISC) to perform its regulatory functions in two primary ways [49,50]. In the first and more common mechanism, when the *miRNA* sequence is nearly fully complementary to the target mRNA, enabling the RISC complex to cleave the target mRNA with the help of nucleic acid exonucleases, and therefore downregulate the expression of the target gene [51]. In the second mechanism, the *miRNA* binds to an incompletely complementary site within the 3′ non-coding region (UTR) of the target gene mRNA, resulting in translational repression without direct cleavage [52]. Although mature *miRNAs* are short, typically consisting of only 20–25 nucleotides, thousands of *miRNA* molecules have been identified in plants, indicating considerable diversity in their sequences.

*miRNA* molecules play central regulatory roles in numerous biological processes, including plant embryonic development [53], vegetative growth [54], and the development of leaves and floral organs [55,56]. *miR172* regulated the expression of floral organ identity gene AP2-like transcription factors through cleavage and translational repression of target gene mRNAs, thereby controlling flowering time and floral organ differentiation in *Arabidopsis thaliana* [57]. In rice, *miR172* shared similar mechanisms with its *Arabidopsis* counterpart, overexpression of the *OsMIR172B* gene in rice led to delayed flowering time, defects in flower and seed development, such as altered number and characteristics of floral organs, reduced seed setting rate and seed weight, suggesting that *miR172* played a significant regulatory role in plant reproductive growth [58]. Moreover, various *miRNAs* have also been found to play key roles in plant disease tolerance and stress response. For instance, *miR169* enhanced the drought tolerance of plants by repressing the expression of the *NUCLEAR FACTOR Y A5* (*NFYA5*) gene; the protein abundance of NFYA5 was increased in *mir169* knockout mutants, while the protein expression level of NFYA5 in *35S::MIR169* overexpression plants was lower than that of the wild type [26].

### 2.2. ta-siRNA Synthesis, Molecular Mechanisms, and Biological Functions

**Figure 2:**
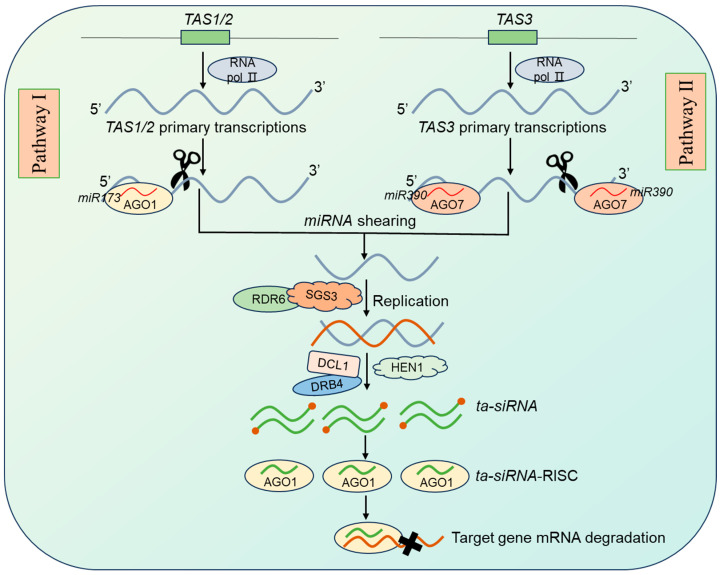
*ta-siRNA* biogenesis and degradation. RNA polymerase II acts on specific *TAS* sites to generate *TAS* primary transcripts, which are recognized and cleaved by specific *miRNAs* (*miR173* or *miR390*). The cleaved *TAS* transcripts further mediate the generation of mature *ta-siRNA*, which then target and inhibit target gene expression. In this figure, black arrows indicate activation processes; red dots represent methylated *ta-siRNAs*; black crosses indicate degraded target mRNAs.

*ta-siRNAs* are another type of *siRNA* molecule found in plants, with a size of 21 nt. Its biogenesis pathway is closely related to the *TAS* (*TRANS-ACTING SMALL RNA*) gene. *TAS* refers to precursor transcripts that give rise to *ta-siRNAs*. Eight *TAS* sites have been identified in *Arabidopsis thaliana*, belonging to four families: *TAS1*, *TAS2*, *TAS3,* and *TAS4*. *miRNAs*-mediated *ta-siRNAs* biogenesis process mainly occurs through two pathways (Figure 2): Pathway I, the *TAS* initial transcripts generated by transcription of the *TAS* sites are cleaved at the 5′ end by a single *miRNA*. Specifically, *miR173* targets *TAS1/2* primary transcripts, and *miR828* targets *TAS4* primary transcripts to mediate the cleavage of the 5′ end of the primary transcripts in the presence of ARGONAUTE 1 (AGO1) protein. With the synergistic action of *RNA-DEPENDENT RNA POLYMERASE 6* (*RDR6*) and gene silencing repressor *SUPPRESSOR OF GENE SILENCING 3* (*SGS3*), the cleaved 3′ terminus can be repurposed as a template to form lengthy double-stranded RNA molecules, which are subsequently recognized by the DCL1-Double-stranded RNA-Binding Protein 4 (DRB4) complex and precisely cleaved into a 21 nt *ta-siRNA*. After HEN1 terminal methylation modification, one of the *siRNA* strands will target and cleave the mRNA of the target gene [30]. Pathway II: The presence of two *miRNA* binding sites on the same *TAS* primary transcript jointly activates the production of *ta-siRNAs*. Specifically, *miR390* targets the two sites at the 5′ and 3′ ends of the *TAS3* primary transcripts, respectively. In *Arabidopsis thaliana*, the 3′ end of the *miR390*-targeted site is cleaved by ARGONAUTE 7 (AGO7), whereas *miR390* at the 5′ end does not act in shearing activity. Subsequently, as in pathway I, a 21-nt *siRNA* is produced when RDR6-synthesized dsRNA is cleaved by DCL4, which ultimately targets and cleaves the mRNA site [30]. The biogenesis of *ta-siRNA* closely links *miRNAs* and *siRNAs*, which were originally thought to act independently. Therefore, some researchers proposed a hierarchical regulatory network that extends from *miRNAs* to *siRNAs* and ultimately affects the target gene. The interplay between *miRNAs* and *siRNAs* may represent a molecular immune system developed by cells during evolution for precise regulation of endogenous gene expression.

*TAS* genes, responsible for the biogenesis of *ta-siRNAs*, are essential in the growth and development of plants. Among the four *TAS* families, *Trans-Acting Small Interfering RNA 3* (*TAS3*) plays a particularly significant regulatory role in plant developmental processes. *TAS3*-derived *ta-siRNAs* target mRNAs of *AUXIN RESPONSE FACTOR* (*ARF*) family members *ARF2*, *ARF3*, and *ARF4* (*tasiR-ARFs*), thereby influencing a variety of growth and developmental processes, including lateral root formation [59], leaf polarity development [60], and floral organ morphogenesis [61]. In 2010, researchers indicated that *miR390* and *TAS3*-derived *ta-siRNAs*, in collaboration with *ARFs*, formed a precise regulatory network that modulated lateral root growth [59]. The initiation of root formation triggered *miR390* induction, leading to the local production of *ta-siRNAs* that downregulated *ARF2*/*3*/*4* and promoted root growth. Furthermore, *ARFs* provided feedback regulation of *miR390* expression, thereby completing an autoregulatory network [59]. Additionally, *ta-siRNAs* were implicated in various regulatory processes, such as plant hormone response [62], cellular morphogenesis [63], and plant secondary metabolism [64].

### 2.3. Hc-siRNA Synthesis, Molecular Mechanisms, and Biological Functions

*Heterochromatic small interfering RNAs* (*hc-siRNAs*) are small RNA molecules that originate from heterochromatin regions of the genome. *Hc-siRNAs* are derived from transposable elements, retrotransposons, repetitive sequences, and intergenic spacer regions; these *siRNAs* are also known as *repeat-associated-siRNAs* (*ra-siRNAs*). They are predominantly 24 nt in length and are the most abundant *siRNAs* in *Arabidopsis*, accounting for over 90% of the *siRNA* population [65].

**Figure 3:**
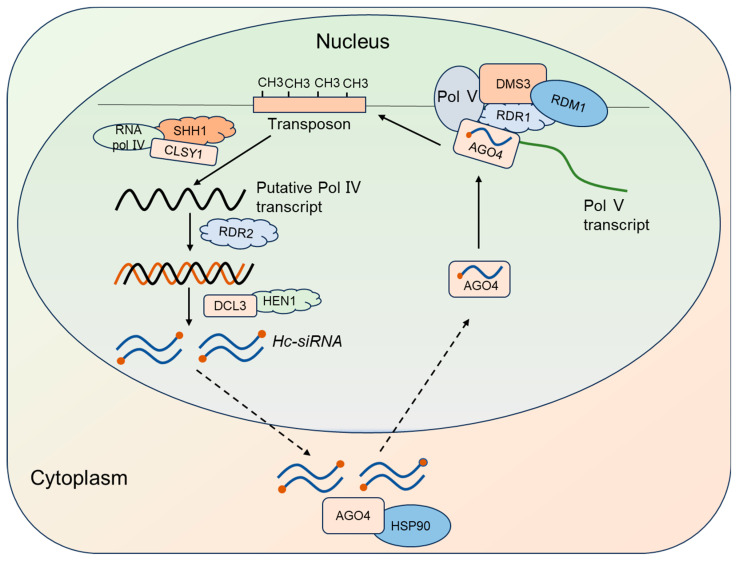
*Hc-siRNA* biogenesis and mechanism. Heterochromatin DNA is transcribed into a single-stranded RNA, which is then replicated by *RDR2* to form double-stranded RNA (dsRNA). This dsRNA is processed by DCL3 and HEN1 to form mature *hc-siRNA*. Then, *hc-siRNA* is exported to the cytoplasm, where it binds to the AGO4 complex. With the assistance of HSP90, *hc-siRNA* is transported back to the nucleus, and mediate heterochromatinization of endogenous repeat sequences or histone methylation modifications. In this figure, black arrows indicate activation processes; red dots represent methylated *hc-siRNAs*.

The biogenesis of *hc-siRNAs* is as follows (Figure 3): heterochromatin DNA is transcribed by Pol IV to produce single-stranded RNA, which is then replicated by *RNA-DEPENDENT RNA POLYMERASE 2* (*RDR2*) to form double-stranded RNA (dsRNA). This dsRNA is processed by *DICER-LIKE 3* (*DCL3*)*,* which cleaves it into 24nt *hc-siRNAs*. Subsequently, the methyltransferase HEN1 modifies the 3′ ends of these *hc-siRNAs* via methylation. Mature *hc-siRNAs* are exported from the nucleus to the cytoplasm, where they bind to the ARGONAUTE 4 (AGO4) complex. With the assistance of HEAT-SHOCK PROTEIN 90 (HSP90), these *hc-siRNAs* are transported back to the nucleus, where they mediate heterochromatinization of endogenous repeat sequences or histone methylation modifications [66].

*Hc-siRNAs* mainly function to mediate cytosine methylation modifications, playing a critical role in RNA-mediated DNA methylation (RdDM) and other epigenetic regulation in *Arabidopsis* [67]. In 2006, studies indicated that 24 nt *hc-siRNAs* could bind to AGO4 to form an RNA-induced transcriptional silencing complex that participated in catalysis, guiding *DOMAINS REARRANGED METHYLTRANSFERASE 2* (*DRM2*) to perform RNA-mediated DNA methylation modifications on the cytosines of homologous genomic sequences [68]. *Hc-siRNAs* also played a crucial role in plant immunity [69]. Research found that the accumulation levels of *hc-siRNAs* could be modulated by pathogen-induced signals, subsequently influencing the plant immune response [69]. For instance, fungal elicitors could trigger alterations in the accumulation of specific *hc-siRNAs*, thereby participating in the regulation of plant defenses against pathogens [70].

### 2.4. nat-siRNA Synthesis, Molecular Mechanisms, and Biological Functions

**Figure 4:**
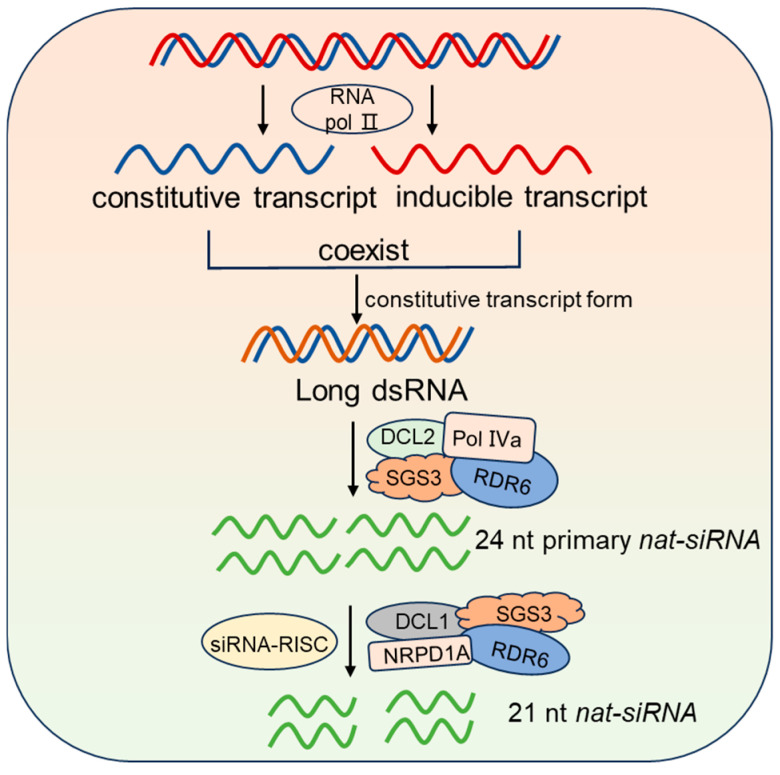
*nat-siRNA* biogenesis. When both constitutive transcripts and inducible transcripts coexist, the constitutive transcript mRNA is replicated to form double-stranded RNA, then it is cleaved by DCL2, Pol IVa, RDR6, and SGS3 to generate 24 nt primary *nat-siRNA*. The cleaved transcript is further processed by DCL1, RDR6, SGS3, and NRPD1A proteins to produce 21 nt *nat-siRNA*. The detailed mechanism remains unclear. In this figure, black arrows indicate activation processes.

In plants, certain noncoding RNA transcripts are reverse-complementary and partially overlap with mRNAs on the opposite strands, with one transcript being constitutively expressed (constitutive transcript) and the other induced under stress conditions. When both transcripts coexist, the constitutively expressed transcript mRNA forms long dsRNA. This dsRNA is then cleaved to generate 24 nt primary *nat-siRNAs* through the combined action of DICER-LIKE 2 (DCL2), Pol IVa, RDR6, and SGS3. These primary *nat-siRNAs* then guide the siRNA-RISC complex to recognize and cleave the constitutively expressed transcript. The cleaved transcript is further processed by DCL1, RDR6, SGS3 and Nuclear RNA Polymerase D 1A (NRPD1A) proteins to produce 21 nt *nat-siRNAs* (Figure 4) [71,72].

Although the detailed mechanism of the process remains unclear, these *nat-siRNAs* have been found to play crucial roles in plant responses to abiotic stresses [72]. Specifically, *nat-siRNAs* were demonstrated to enhance plant salt tolerance by modulating the expression of the *P5CDH* gene [72]. However, further exploration is needed to confirm the function of *nat-siRNA* in reproductive processes.

## 3. Small RNAs Regulate the Process of Plant Megasporogenesis

During the early stages of plant ovule sexual reproduction development, megasporogenesis requires the specialization of a somatic cell in the L2 layer into an MMC, which then undergoes one round of meiosis to form an FM. In plant megasporogenesis, the FM is the haploid product of meiosis that undergoes mitotic divisions to form the mature embryo sac. The differentiation of the MMC from somatic cells marks the initiation of the female germ line in flowering plants [1]. The integrity of megasporogenesis is critical for the development of the female reproductive unit and the completion of double fertilization. Small RNAs, as pivotal negative regulators in eukaryotic gene expression, play a crucial role in orchestrating MMC differentiation and its subsequent progression into meiosis, representing a fascinating frontier in biological research. What are the underlying molecular mechanisms that govern their translocation within sporophyte and gametophyte tissues? Which coding genes and small RNAs are implicated in these processes? These questions remain largely unanswered, with research only beginning to uncover the complexity of this biological phenomenon.

**Figure 5:**
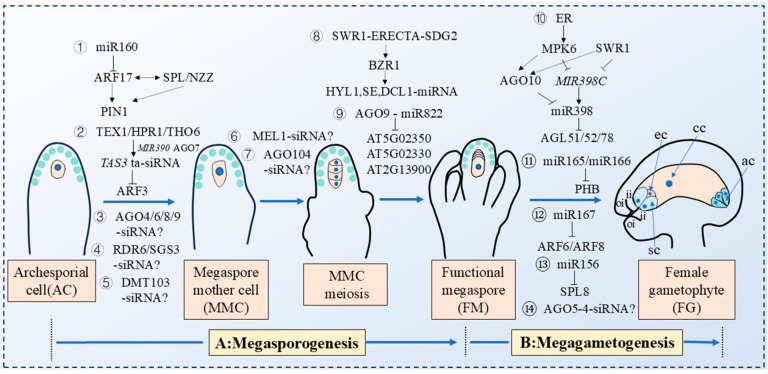
Small RNAs are involved in the regulation of plant female gametophyte development. A: Megasporogenesis. The small RNAs involved in this process are as follows: (1) The target gene *ARF17* of *miR160* interacted genetically with the *SPL/NZZ* gene, and they ensured the formation of only one MMC in the ovule subepidermal layer by regulating the expression domain of PIN1. (2) The transcription and production of *TAS3 ta-siRNA* were promoted by TERMINAL EXON1 (TEX1), etc., *TAS3 ta-siRNA* targeted inhibition of ARF3 and regulated MMC specialization. (3) AGO family members ARGONAUTE 4/6/8/9 (AGO4/6/8/9) mediated MMC establishment through *siRNAs* pathway. (4) *siRNAs* regulated MMC specialization by moving between cells through RDR6 and SGS3. (5) *DMT103* encoded methyltransferase, which was involved in RNA-mediated DNA methylation and MMC formation together with *siRNAs* and other proteins. (6) MEL1 bound to *siRNAs* to regulate the entry of MMC into meiosis. (7) ARGONAUTE 104 (AGO104) participated in the meiosis process of MMC through a certain *siRNA*-mediated gene regulatory network. (8) As the downstream effector molecule of SWR1-ERECTA-SDG2 signaling module, BZR1 transcription factor directly activated the expression of *HYL1*, *SE* and *DCL1* genes in the *miRNA* processing complex, and synergistically regulated the process of *Arabidopsis* megasporogenesis. (9) The AGO9-*miR822* signaling pathway regulated unicellular development by limiting the survival of meiosis-derived cells to a single megaspore. B: Megagametogenesis. The small RNAs involved in this process are as follows: (10) SWR1 and ERECTA signaling pathways promoted the expression of AGO10 at the chalazal, localized and limited *miR398* to the chalazal region and could not be transferred to the female gametophyte, ensuring that the target gene *AGAMOUS-LIKE 51/52/78* (*AGL51/52/78*) of *miR398* were normally expressed in the embryo sac. (11) *miR165/166* affected the integument development of *Arabidopsis* ovules by regulating the expression of *PHABULOSA* (*PHB*) gene. (12) *miR167* negatively regulated the target genes of *AUXIN RESPONSE FACTOR 6/8* (*ARF6*/*8*), the construction of mARF6 transgenic plants and *mir167* mutants would hinder the development of inner and outer integument, resulting in the exposure of embryo sac and affecting the fertility of female gametes. (13) *miR156* targeted the mRNA expressed by *SQUAMOSA PROMOTER BINDING PROTEIN-LIKE 8* (*SPL8*) gene and regulated the development of male and female gametophytes in *Arabidopsis thaliana*. (14) The insertion mutation of *ago5-4* produced a semi-dominant *ARGONAUTE 5* (*AGO5*) gene, which inhibited the initiation of female gametophyte development, caused the FM to stagnate at FG1 stage and could not undergo mitosis, and the female gametophyte could not form normally. AC: Archesporial cell; MMC: Megaspore mother cell; FM: Functional megaspore; FG: Female gametophyte; ii: inner integument; oi: outer integument; ec: egg cell; sc: synergid cell; cc: central cell: ac: antipodal cell; “?”: There is no specific discovery of what kind of small RNA.

In 2022, Huang Jian et al. discovered that *ARF17*, a target gene of *miR160*, genetically interacted with the *SPOROCYTELESS/NOZZLE* (*SPL/NZZ*) gene to maintain the maximum auxin concentration at the ovule apex by regulating the expression domain of the auxin transporter PIN-FORMED 1 (PIN1), this regulation created a restrictive signal that ensured the formation of a single MMC in the ovule subepidermal layer [73]. Mutation of the *miR160a* resulted in a high ovule abortion rate (66.1%), with multiple MMC-like cells forming in ovules during megasporogenesis (Table S2). Additionally, *pARF17::mARF17-GFP* transgenic plants displayed multiple MMC phenotypes, indicating the importance of *miR160*-mediated regulation of *ARF17* in restricting MMC formation to a single cell in *Arabidopsis thaliana* ovules (Figure 5; Table S1) [73]. Another *miRNA* gene implicated in megasporogenesis is *MIR822*. In 2024, researchers proposed that the AGO9-*miR822* signaling pathway regulated monosporic development by limiting the survival of meiosis-derived cells to a single megaspore during *Arabidopsis* megasporogenesis (Table S1) [74]. The *MIR822* gene was found to be specifically expressed throughout ovule development. In the *mir822* mutants, the early process of megaspore formation closely resembled that of the wild type. Specifically, the ovule primordium cell developed into a single MMC. This MMC then underwent meiosis, giving rise to four haploid megaspores. However, the subsequent formation of an FM was accompanied by a failure of the other three megaspores to degrade (*mir822-1*: 37.73%; *mir822-2*: 32.11%) in *mir822* mutants, and these non-degraded megaspores could obtain FM identity. These additional FMs formed two nuclei by mitosis in the FG4 stage, but they stagnated at the two-nucleus stage, eventually leading to partial ovule abortion (Table S2) [74]. Further studies identified three target genes of *MIR822*—*At5g02350*, *At5g02330,* and *At2g13900*, which encode cysteine/histidine-rich domain proteins. Overexpression of these genes resulted in the abortion of unfertilized ovules prior to seed formation, phenocopying the *mir822* mutant phenotype (Figure 5) [74]. In 2023, a study revealed the pivotal role of the *SWI2/SNF2-RELATED 1* (*SWR1*)*–ERECTA*(ER)*-SET DOMAIN GROUP 2* (*SDG2*) signaling module in regulating female germ cell specialization in *Arabidopsis* [75]. In *arp6-er-sdg2* triple mutants, multiple MMC-like cells abnormally formed and entered meiosis, leading to the production of multiple FMs and severe disruptions during the megasporogenesis stage. Further investigation indicated that the *BRASSINAZOLE-RESISTANT 1* (*BZR1*) transcription factor, a downstream molecule of the *SWR1-ERECTA-SDG2* signaling module, directly activates the expression of *SE*, *HYL1*, and *DCL1*, key genes in the *miRNAs* processing complex, thereby synergistically regulating the development of female gametophytes in *Arabidopsis* (Figure 5) [75]. These findings clarified the important function of the *SWR1-ERECTA-SDG2* signaling module in plant reproductive development and provided new insights into the molecular mechanisms underlying plant germ cell fate specialization.

Moreover, *ta-siRNAs* have also been revealed to play key roles in the early development of female gametophytes, particularly in the formation of MMC. In 2010, researchers, including Olmedo-Monfil and Duran-Figueroa, found that *ta-siRNAs* function through a non-cell autonomous silencing mechanism to mediate the regulation of MMC specialization in *Arabidopsis thaliana*. This process was dependent on the AGO9 protein, which facilitated the signaling pathway that guided the silencing of target genes, thereby influencing MMC development [76,77]. *ta*-*siRNAs* preferentially bind to AGO9 protein in L1 layer cells and are then transported to the L2 layer gametophyte tissue in ovules. These small RNAs mediated AGO9-induced silencing of transposon TEs, preventing the transformation of somatic cells adjacent to the MMC in the L2 layer of the ovule into MMC. In contrast, the *ago9* mutation disrupted the biological function of these *ta-RNAs*, leading to the formation of multiple MMCs in the ovule (Figure 5) [76,77]. Further research revealed that *ta-siRNAs*, acting as signaling molecules, facilitated intercellular movement by inducing *RDR6* transcription and leveraging *SGS3*. Mutations that impaired the functions of *rdr6* and *sgs3* resulted in the transformation of L2 somatic cells into MMC-like cells [78,79,80]. In addition, mutations in other AGO family members (*AGO4*, *AGO6*, and *AGO8*) in *Arabidopsis* also led to the formation of multiple MMC-like cells (Figure 5) [81].

The TEX1 protein was a key component of the TREX complex, and research revealed that the TREX complex was involved in the production of *ta-siRNAs* in plants [82]. In 2017, our research found that the *tex1* mutants resulted in the formation of multiple abnormally enlarged cells in the ovule primordium, some of which exhibited molecular characteristics of MMC, but only one MMC entered meiosis [83]. TEX1 protein was specifically expressed in the L1 layer somatic cells of the ovule during the MMC stage. TEX1 protein promoted the transcription and production of *TAS3 ta-siRNA* in L1 layer cells, which then targeted and suppressed the transcription and expression of ARF3 in the L1 layer-specific region of the ovule, thus regulating MMC occurrence (Table S1). Mutations in other TREX complex components (*hprl* and *tho6* mutants), *tas3* mutants in the *ta-siRNA* synthesis pathway, and *TAS3 ta-siRNA* insensitive transgenic mutants (*pARF3::ARF3m-GFP*), similarly resulted in the production of multiple MMCs in the ovules (Figure 5; Table S2) [83]. In 2020, Su et al. further found that mutations in other genes involved in the *tasiR-ARF* biosynthesis, such as *mir390* and *ago7*, also led to the formation of multiple MMCs. These abnormally enlarged MMC-like cells possessed the molecular characteristics of MMC but failed to enter meiosis (Table S2) [84]. Using promoter-fusion expression vectors and *TAS3 ta-siRNA* insensitive mutants (*pSPL::ARF3m-mCitrine* and *pWRKY28::ARF3m-mCitrine*), it was observed that the expression of *ARF3m* in L2 subcuticular cells led to the formation of multiple MMCs in the ovule primordium, while *ARF3m* expression in the epidermal cell layer did not disrupt ovule development (Figure 5) [84]. This indicates that plants have established complex regulatory networks in different ovule cell layers to ensure the formation of only one MMC in subcutaneous cells [84].

The *MEIOSIS ARRESTED AT LEPTOTENE 1* (*MEL1*) gene in rice encoded a member of the AGO protein family and was homologous to *Arabidopsis* AGO5. *siRNAs* specifically bound to the MEL1 protein, which was expressed in the cytoplasm of the MMC, thereby regulating MMC development in rice [85]. However, the specific *siRNA* molecules involved in this regulatory process require further investigation. A loss-of-function mutation in *mel1* led to abnormal meiosis in the MMC within the ovule, preventing the normal formation of the female gametophyte and thereby affecting plant sexual reproduction (Figure 5) [86]. In maize, *AGO104* also encoded a crucial AGO protein, which participated in the development of MMC through a small RNA-mediated gene regulatory network. In *ago104* mutants, the MMC developed normally but failed to further enter meiosis, resulting in abnormal female gametophyte development [87]. Unexpectedly, the AGO104 protein was specifically expressed in somatic cells surrounding the MMC. This suggested that the subepidermal cells around the MMC produced a mobile signal that was dependent on AGO104. This signal, in turn, influenced the entry of the MMC into meiosis (Figure 5) [87]. Additionally, *siRNAs*, in conjunction with the *DMT103* gene in maize, also played a crucial role in the MMC specialization process [88]. *DMT103* encoded a methyltransferase homologous to the *DOMAINS REARRANGED METHYL TRANSFERASE* (*DRMs*) gene in *Arabidopsis* and was involved in RNA-mediated DNA methylation with *siRNAs* and other proteins [88]. Garcia-Aguilar et al. demonstrated that down-regulation of *DMT103* expression resulted in the expansion of adjacent cells to the MMC in the subepidermal layer of the maize embryo sac, some of which exhibited characteristics of MMC (Figure 5) [89].

## 4. Small RNAs Regulate the Process of Plant Megagametogenesis

The late stages of female gametophyte development involve numerous fundamental biological phenomena, including nuclear migration and fusion, vacuole formation, cell polarity establishment, apoptosis, asymmetric division, and cell fate determination. However, our recognition of the genes that regulate these cellular events, particularly small RNAs, and their potential molecular mechanisms remains limited [90,91]. In *Arabidopsis*, the formation of an embryo sac with eight nuclei requires the FM to undergo three rounds of mitosis. The megagametogenesis can be categorized into seven stages (FG1-FG7) based on the number of nuclei in the embryo sac, vacuole formation, and the distribution of cell types. At the FG1 stage, the FM elongates along the chalazal-micropyle axis, and its volume gradually increases to form an oval cell. During the FG2 stage, the FM undergoes the first nuclear mitosis to form two nuclei; subsequently, a large number of small vacuoles are formed within the embryo sac and aggregate to form a central large vacuole, which pushes the two nuclei towards the micropylar and the chalazal (FG3 stage). At the FG4 stage, with the development of the ovule, the embryo sac undergoes the second mitosis to form four nuclei. In the FG5 stage, the third mitosis occurs, resulting in an eight-nucleated embryo sac; cell walls begin to form, and the inner and outer integument rapidly wrap the nucleus to complete the process of cellularization. During the FG6 stage, one nucleus from each end of the embryo sac moves to the center, and the polar nuclei fuse, forming the characteristic “seven-cell eight-nucleate” structure. Finally, three antipodal cells degenerate, and the mature embryo sac, composed of one central cell, one egg cell, and two synergid cells, forms the female reproductive unit (FG7 stage) [92].

As significant negative regulators of plant reproductive development, how do small RNAs participate in the complex regulation of integument formation and ovule morphogenesis? In 2006, Wu et al. revealed the molecular mechanism of *MIR167* regulating the development of female gametophytes in *Arabidopsis thaliana* [93]. *ARF6* and *ARF8*, which encode auxin response factors, were targeted by *miR167* and exhibited functional redundancy. By constructing *ARF6*-insensitive transgenic lines (*pARF6::ARF6m-GFP*), which disrupted the complementary base pairing between *miR167* and *ARF6* target sites, researchers observed that cell morphology and arrangement in the ovule remained normal during late female gametophyte development, while the growth of both inner and outer integuments was halted. This prevented the nucellus from being fully enclosed, leaving the embryo sac exposed and negatively impacting the fertility of the female gametophyte [93]. The arrested integument development in *pARF6::ARF6m-GFP* transgenic plants indicated that *miR167* functions to inhibit *ARF6* expression in the integument. The consequent abnormal development of the inner and outer integuments disrupted embryo sac development, which was the primary reason for female gametophyte abortion (Figure 5) [93]. In 2019, researchers utilized CRISPR-Cas9 gene editing technology to construct *mir167* mutants to further explore the function of *miR167* in plant embryo sac development [94]. The *mir167* mutations exhibited delayed flowering time, defects in anther dehiscence, and failure of timely pollen release. The embryo sac protruded from the micropylar end and stalled at various developmental stages, preventing the formation of female reproductive units. It ultimately resulted in smaller and shriveled siliques and a significantly reduced seed setting rate (Table S2) [94]. In vitro pollen germination experiments indicated that the developmental defects of anther, ovule, and seed setting rate in *mir167* mutants were due to the overexpression of its target genes, *ARF6* and *ARF8*. Expression pattern analysis revealed that the *MIR167A* gene was expressed in anther wall cells and ovule epidermal cells, suggesting that *MIR167A* is possibly involved in anther dehiscence and integument regulation (Figure 5; Table S1) [94]. Furthermore, *miR156* has been found to regulate the female gametophytes’ development by targeting the mRNA expressed by the *SPL8* gene in *Arabidopsis thaliana* (Figure 5; Table S1) [95]. *MIR165/166*, another class of small RNA genes coordinated with their target gene *PHB* and AGO10 to accurately regulate embryonic development in a timely and spatially specific manner in *Arabidopsis*. Additionally, *miR165*/*166* also influenced ovule development by regulating *PHB* expression in the female reproductive development stage (Table S1). In *phb* mutants, the growth of the ovule’s outer integument was inhibited, leaving the embryo sac exposed. This abnormality resulted in defective ovule development and ultimately the inability to carry out normal double fertilization (Figure 5) [96].

In 2012, Mattew et al. identified a semi-dominant insertion mutant of *ago5-4*, different from the mechanism of AGO9 binding to 24 nt *siRNAs* in the epidermal cell layer to inhibit the formation of multiple MMCs in a non-cell-autonomous manner. The specialization of MMC and the formation of FM in the *ago5-4* semi-dominant insertion mutants were normal during megasporogenesis. Further research indicated that the insertion of *ago5-4* produced a semi-dominant *AGO5* gene, which inhibited the initiation of megagametogenesis, causing the FM to stagnate at the FG1 stage and preventing normal female gametophyte development, thereby reducing the seed setting rate [97]. It was possible that the *ago5-4* mutants may produce a truncated, tissue-specific AGO5 protein that affected the binding efficiency of small RNAs. This alteration led to the de-repression of their complementary targets and disrupted a novel pathway necessary for transitioning to the gametophyte stage (Figure 5) [97]. In 2021, our group elucidated the molecular mechanism by which the chromatin remodeling complex *SWR1* and the *ERECTA* receptor kinase signaling pathway regulated the biosynthesis of *miR398* in a temporal and spatial manner, thereby modulating the ovule development of *Arabidopsis thaliana* [98]. *pri-miR398c* was initially transcribed in female gametophytes and then transported to ovule sporophytes for further processing, indicating a strict regulation of *miR398*’s spatial distribution. The *SWR1* and *ERECTA* signaling pathways also promoted the expression of AGO10 at the chalazal, which localized and limited *miR398* to the region, preventing its transfer to the female gametophyte. This regulation ensured the proper expression of *miR398* target gene *AGL51/52/78* in the embryo sac, which not only promoted the normal development of the female gametophyte tissue, but also played key roles in the proper formation of sporophyte integument tissue (Figure 5; Table S1) [98].

## 5. Conclusions and Prospect

As significant negative regulators, small RNAs have also been reported to participate in the regulation of numerous core biological regulatory events in plants. Although our comprehension of the regulatory functions of small RNAs in various processes has progressively expanded, including organ development [20,21,22], vegetative growth [23,24], disease resistance, and stress tolerance [25,26,27,28,29], research on small RNAs in plant reproductive development, particularly in the development of female gametophytes, has yet to be fully elucidated. There are two primary reasons for this gap: First, compared to male gametophytes, female gametophytes are deeply embedded within the carpel and ovary tissues and exist in limited numbers, both of which hinder experimental observation and analysis. Second, mature small RNAs function as repressive regulators that move non-cell-autonomously between sporophytic and gametophytic tissues, and their dynamic spatiotemporal expression patterns further complicate functional studies during reproductive development [80,98]. In this review, we have systematically summarized recent advances in the post-transcriptional regulatory mechanisms mediated by small RNAs during various biogenetic processes in plants. We focused particularly on their roles in reprogramming somatic cells into reproductive cell fates and delineating the regulatory networks governing female gametophyte development.

In recent years, with the advancement of various modern biological technologies, such as CRISPR-Cas9 gene editing technology, transcriptome sequencing and other emerging biotechnologies, we are now equipped with laser-capture microdissection (LCM) or tissue-cell-specific omics techniques, along with other sophisticated research methods, to construct models of *miRNA*-target gene interactions in mediating important biological processes and reveal their molecular regulatory networks [99,100]. Combined with the functional genomics approaches, the mechanism of small RNAs and their target genes in regulating plant growth, development, and stress response has been systematically and deeply analyzed. Furthermore, the application of CRISPR-Cas9 technology to engineer small RNA-deficient mutants offers new opportunities to characterize the regulatory roles of small RNAs in plant female gametophyte development. These insights will not only deepen our understanding of small RNAs in fundamental biological problems, but also provide a theoretical basis for future crop improvement through molecular design or special agronomic cultivation [101,102].

However, there are still some shortcomings. Studies have pointed out that small RNAs not only transmit short distances between cells via plasmodesmata, but also achieve long-distance transport through sieve tubes [103]. Whether there are differences in the specific mechanisms of small RNA molecules silencing target genes in the production site and when transported to remote sites requires further clarification. It has also been noted that many small RNAs are involved in both mRNA cleavage and translation inhibition in plants, such as *miR156/157* and *miR398* [29,104]. However, the balance and molecular mechanism between these two modes of action remain unclear, necessitating further investigation. Additionally, while plant *miRNAs* have been well characterized during vegetative growth, the genetic basis and molecular mechanism for regulating the specification of MMC, the apoptosis and degradation of three haploid megaspores, the survival of only one FM, the ovule integuments development, and the normal progression of the embryo sac, remains largely enigmatic. Therefore, there exists a pressing need to discover more small RNA genes that play vital roles during the processes of megasporogenesis and megagametogenesis in plants.

## Supplementary Materials

The following supporting information can be downloaded at: https://www.mdpi.com/article/10.3390/plants14091286/s1. Table S1. Classifications and functions of small RNAs in female gametophyte development. Table S2. Key mutants and phenotypes in small RNA-mediated female gametophyte development.

## References

[1] Yang W.C., Shi D.Q., Chen Y.H. (2010). Female gametophyte development in flowering plants. Annu. Rev. Plant Biol..

[2] Drews G.N., Koltunow A.M. (2011). The female gametophyte. Arabidopsis Book..

[3] Chevalier É., Loubert-Hudon A., Zimmerman E.L., Matton D.P. (2011). Cell-cell communication and signalling pathways within the ovule: From its inception to fertilization. New Phytol..

[4] Hanyang C., Suzhuo M., Han S. (2022). Positional signals establishment in the regulation of female germline specification. Seed Biol..

[5] Reinhart B.J., Weinstein E.G., Rhoades M.W., Bartel B., Bartel D.P. (2002). MicroRNAs in plants. Genes. Dev..

[6] Llave C., Xie Z., Kasschau K.D., Carrington J.C. (2002). Cleavage of Scarecrow-like mRNA targets directed by a class of Arabidopsis miRNA. Science.

[7] Park W., Li J., Song R., Messing J., Chen X. (2002). CARPEL FACTORY, a Dicer homolog, and HEN1, a novel protein, act in microRNA metabolism in *Arabidopsis thaliana*. Curr. Biol..

[8] Hsieh L.C., Lin S.I., Shih A.C., Chen J.W., Lin W.Y., Tseng C.Y., Li W.H., Chiou T.J. (2009). Uncovering small RNA-mediated responses to phosphate deficiency in Arabidopsis by deep sequencing. Plant Physiol..

[9] Song L., Axtell M.J., Fedoroff N.V. (2010). RNA secondary structural determinants of miRNA precursor processing in Arabidopsis. Curr. Biol..

[10] Sunkar R., Zhu J.K. (2004). Novel and stress-regulated microRNAs and other small RNAs from Arabidopsis. Plant Cell.

[11] Xie Z. (2010). Piecing the puzzle together: Genetic requirements for miRNA biogenesis in *Arabidopsis thaliana*. Methods Mol. Biol..

[12] Li Y.F., Zheng Y., Addo-Quaye C., Zhang L., Saini A., Jagadeeswaran G., Axtell M.J., Zhang W., Sunkar R. (2010). Transcriptome-wide identification of microRNA targets in rice. Plant J. Cell Mol. Biol..

[13] Meng Y., Chen D., Ma X., Mao C., Cao J., Wu P., Chen M. (2010). Mechanisms of microRNA-mediated auxin signaling inferred from the rice mutant osaxr. Plant Signal Behav..

[14] Sunkar R., Girke T., Jain P.K., Zhu J.K. (2005). Cloning and characterization of microRNAs from rice. Plant Cell.

[15] Mica E., Gianfranceschi L., Pè M.E. (2006). Characterization of five microRNA families in maize. J. Exp. Bot..

[16] Yao Y., Guo G., Ni Z., Sunkar R., Du J., Zhu J.K., Sun Q. (2007). Cloning and characterization of microRNAs from wheat (*Triticum aestivum* L.). Genome Biol..

[17] Li B., Yin W., Xia X. (2009). Identification of microRNAs and their targets from Populus euphratica. Biochem. Biophys. Res. Commun..

[18] Feng J., Wang K., Liu X., Chen S., Chen J. (2009). The quantification of tomato microRNAs response to viral infection by stem-loop real-time RT-PCR. Gene.

[19] Gu M., Xu K., Chen A., Zhu Y., Tang G., Xu G. (2010). Expression analysis suggests potential roles of microRNAs for phosphate and arbuscular mycorrhizal signaling in Solanum lycopersicum. Physiol. Plant.

[20] Gutierrez L., Bussell J.D., Pacurar D.I., Schwambach J., Pacurar M., Bellini C. (2009). Phenotypic plasticity of adventitious rooting in Arabidopsis is controlled by complex regulation of AUXIN RESPONSE FACTOR transcripts and microRNA abundance. Plant Cell.

[21] Wang J.W., Wang L.J., Mao Y.B., Cai W.J., Xue H.W., Chen X.Y. (2005). Control of root cap formation by MicroRNA-targeted auxin response factors in Arabidopsis. Plant Cell.

[22] Pulido A., Laufs P. (2010). Co-ordination of developmental processes by small RNAs during leaf development. J. Exp. Bot..

[23] Schmid M., Uhlenhaut N.H., Godard F., Demar M., Bressan R., Weigel D., Lohmann J.U. (2003). Dissection of floral induction pathways using global expression analysis. Development.

[24] Millar A.A., Gubler F. (2005). The Arabidopsis GAMYB-like genes, MYB33 and MYB65, are microRNA-regulated genes that redundantly facilitate anther development. Plant Cell.

[25] Wang M., Wang Q., Zhang B. (2013). Response of miRNAs and their targets to salt and drought stresses in cotton (*Gossypium hirsutum* L.). Gene.

[26] Du Q., Zhao M., Gao W., Sun S., Li W.X. (2017). microRNA/microRNA* complementarity is important for the regulation pattern of NFYA5 by miR169 under dehydration shock in Arabidopsis. Plant J. Cell Mol. Biol..

[27] Gupta O.P., Meena N.L., Sharma I., Sharma P. (2014). Differential regulation of microRNAs in response to osmotic, salt and cold stresses in wheat. Mol. Biol. Rep..

[28] Liang G., He H., Yu D. (2012). Identification of nitrogen starvation-responsive microRNAs in *Arabidopsis thaliana*. PLoS ONE.

[29] Sunkar R., Kapoor A., Zhu J.K. (2006). Posttranscriptional induction of two Cu/Zn superoxide dismutase genes in Arabidopsis is mediated by downregulation of miR398 and important for oxidative stress tolerance. Plant Cell.

[30] Chen X. (2009). Small RNAs and their roles in plant development. Annu. Rev. Cell Dev. Biol..

[31] Chen X. (2005). MicroRNA biogenesis and function in plants. FEBS Lett..

[32] Kim Y.J., Zheng B., Yu Y., Won S.Y., Mo B., Chen X. (2011). The role of Mediator in small and long noncoding RNA production in *Arabidopsis thaliana*. Embo J..

[33] Ambros V., Chen X. (2007). The regulation of genes and genomes by small RNAs. Development.

[34] Lee Y., Ahn C., Han J., Choi H., Kim J., Yim J., Lee J., Provost P., Rådmark O., Kim S. (2003). The nuclear RNase III Drosha initiates microRNA processing. Nature.

[35] Zhao Y., Yu Y., Zhai J., Ramachandran V., Dinh T.T., Meyers B.C., Mo B., Chen X. (2012). The Arabidopsis nucleotidyl transferase HESO1 uridylates unmethylated small RNAs to trigger their degradation. Curr. Biol..

[36] Jones-Rhoades M.W., Bartel D.P., Bartel B. (2006). MicroRNAS and their regulatory roles in plants. Annu. Rev. Plant Biol..

[37] Lee Y., Kim M., Han J., Yeom K.H., Lee S., Baek S.H., Kim V.N. (2004). MicroRNA genes are transcribed by RNA polymerase II. Embo J..

[38] Grigg S.P., Canales C., Hay A., Tsiantis M. (2005). SERRATE coordinates shoot meristem function and leaf axial patterning in Arabidopsis. Nature.

[39] Han M.H., Goud S., Song L., Fedoroff N. (2004). The Arabidopsis double-stranded RNA-binding protein HYL1 plays a role in microRNA-mediated gene regulation. Proc. Natl. Acad. Sci. USA.

[40] Schauer S.E., Jacobsen S.E., Meinke D.W., Ray A. (2002). DICER-LIKE1: Blind men and elephants in Arabidopsis development. Trends Plant Sci..

[41] Vazquez F., Gasciolli V., Crété P., Vaucheret H. (2004). The nuclear dsRNA binding protein HYL1 is required for microRNA accumulation and plant development, but not posttranscriptional transgene silencing. Curr. Biol..

[42] Yang L., Liu Z., Lu F., Dong A., Huang H. (2006). SERRATE is a novel nuclear regulator in primary microRNA processing in Arabidopsis. Plant J. Cell Mol. Biol..

[43] Yu B., Bi L., Zheng B., Ji L., Chevalier D., Agarwal M., Ramachandran V., Li W., Lagrange T., Walker J.C. (2008). The FHA domain proteins DAWDLE in Arabidopsis and SNIP1 in humans act in small RNA biogenesis. Proc. Natl. Acad. Sci. USA.

[44] Kurihara Y., Watanabe Y. (2004). Arabidopsis micro-RNA biogenesis through Dicer-like 1 protein functions. Proc. Natl. Acad. Sci. USA.

[45] Li J., Yang Z., Yu B., Liu J., Chen X. (2005). Methylation protects miRNAs and siRNAs from a 3’-end uridylation activity in Arabidopsis. Curr. Biol..

[46] Yu B., Yang Z., Li J., Minakhina S., Yang M., Padgett R.W., Steward R., Chen X. (2005). Methylation as a crucial step in plant microRNA biogenesis. Science.

[47] Park M.Y., Wu G., Gonzalez-Sulser A., Vaucheret H., Poethig R.S. (2005). Nuclear processing and export of microRNAs in Arabidopsis. Proc. Natl. Acad. Sci. USA.

[48] Ramachandran V., Chen X. (2008). Degradation of microRNAs by a family of exoribonucleases in Arabidopsis. Science.

[49] Baumberger N., Baulcombe D.C. (2005). Arabidopsis ARGONAUTE1 is an RNA Slicer that selectively recruits microRNAs and short interfering RNAs. Proc. Natl. Acad. Sci. USA.

[50] Qi Y., Denli A.M., Hannon G.J. (2005). Biochemical specialization within Arabidopsis RNA silencing pathways. Mol. Cell.

[51] Baulcombe D. (2004). RNA silencing in plants. Nature.

[52] Bagga S., Bracht J., Hunter S., Massirer K., Holtz J., Eachus R., Pasquinelli A.E. (2005). Regulation by let-7 and lin-4 miRNAs results in target mRNA degradation. Cell.

[53] Willmann M.R., Mehalick A.J., Packer R.L., Jenik P.D. (2011). MicroRNAs regulate the timing of embryo maturation in Arabidopsis. Plant Physiol..

[54] Liu Q., Yao X., Pi L., Wang H., Cui X., Huang H. (2009). The ARGONAUTE10 gene modulates shoot apical meristem maintenance and establishment of leaf polarity by repressing miR165/166 in Arabidopsis. Plant J. Cell Mol. Biol..

[55] Rubio-Somoza I., Zhou C.M., Confraria A., Martinho C., von Born P., Baena-Gonzalez E., Wang J.W., Weigel D. (2014). Temporal control of leaf complexity by miRNA-regulated licensing of protein complexes. Curr. Biol..

[56] Chen X. (2004). A microRNA as a translational repressor of APETALA2 in Arabidopsis flower development. Science.

[57] Jung J.H., Seo Y.H., Seo P.J., Reyes J.L., Yun J., Chua N.H., Park C.M. (2007). The GIGANTEA-regulated microRNA172 mediates photoperiodic flowering independent of CONSTANS in Arabidopsis. Plant Cell.

[58] Zhu Q.H., Upadhyaya N.M., Gubler F., Helliwell C.A. (2009). Over-expression of miR172 causes loss of spikelet determinacy and floral organ abnormalities in rice (*Oryza sativa*). BMC Plant Biol..

[59] Marin E., Jouannet V., Herz A., Lokerse A.S., Weijers D., Vaucheret H., Nussaume L., Crespi M.D., Maizel A. (2010). miR390, Arabidopsis TAS3 tasiRNAs, and their AUXIN RESPONSE FACTOR targets define an autoregulatory network quantitatively regulating lateral root growth. Plant Cell.

[60] Garcia D., Collier S.A., Byrne M.E., Martienssen R.A. (2006). Specification of leaf polarity in Arabidopsis via the trans-acting siRNA pathway. Curr. Biol..

[61] Liu X., Dinh T.T., Li D., Shi B., Li Y., Cao X., Guo L., Pan Y., Jiao Y., Chen X. (2014). AUXIN RESPONSE FACTOR 3 integrates the functions of AGAMOUS and APETALA2 in floral meristem determinacy. Plant J. Cell Mol. Biol..

[62] Yoon E.K., Yang J.H., Lim J., Kim S.H., Kim S.K., Lee W.S. (2010). Auxin regulation of the microRNA390-dependent transacting small interfering RNA pathway in Arabidopsis lateral root development. Nucleic Acids Res..

[63] Fahlgren N., Montgomery T.A., Howell M.D., Allen E., Dvorak S.K., Alexander A.L., Carrington J.C. (2006). Regulation of AUXIN RESPONSE FACTOR3 by TAS3 ta-siRNA affects developmental timing and patterning in Arabidopsis. Curr. Biol..

[64] Mohd Zahid N.I.I., Syed Othman S.M.I., Mustaffa A.F., Ismail I., Che-Othman M.H. (2024). Fine-tuning plant valuable secondary metabolite biosynthesis via small RNA manipulation: Strategies and potential. Planta.

[65] Castel S.E., Martienssen R.A. (2013). RNA interference in the nucleus: Roles for small RNAs in transcription, epigenetics and beyond. Nat. Rev. Genet..

[66] Xie Z., Jia G., Ghosh A. (2012). Small RNAs in Plants. MicroRNAs in Plant Development and Stress Responses.

[67] Liu W., Duttke S.H., Hetzel J., Groth M., Feng S., Gallego-Bartolome J., Zhong Z., Kuo H.Y., Wang Z., Zhai J. (2018). RNA-directed DNA methylation involves co-transcriptional small-RNA-guided slicing of polymerase V transcripts in Arabidopsis. Nat. Plants.

[68] Qi Y., He X., Wang X.J., Kohany O., Jurka J., Hannon G.J. (2006). Distinct catalytic and non-catalytic roles of ARGONAUTE4 in RNA-directed DNA methylation. Nature.

[69] Kong X., Yang M., Le B.H., He W., Hou Y. (2022). The master role of siRNAs in plant immunity. Mol. Plant Pathol..

[70] Medina C., da Rocha M., Magliano M., Raptopoulo A., Marteu N., Lebrigand K., Abad P., Favery B., Jaubert-Possamai S. (2018). Characterization of siRNAs clusters in *Arabidopsis thaliana* galls induced by the root-knot nematode Meloidogyne incognita. BMC Genom..

[71] Pikaard C.S., Haag J.R., Ream T., Wierzbicki A.T. (2008). Roles of RNA polymerase IV in gene silencing. Trends Plant Sci..

[72] Borsani O., Zhu J., Verslues P.E., Sunkar R., Zhu J.K. (2005). Endogenous siRNAs derived from a pair of natural cis-antisense transcripts regulate salt tolerance in Arabidopsis. Cell.

[73] Huang J., Zhao L., Malik S., Gentile B.R., Xiong V., Arazi T., Owen H.A., Friml J., Zhao D. (2022). Specification of female germline by microRNA orchestrated auxin signaling in Arabidopsis. Nat. Commun..

[74] Tovar-Aguilar A., Grimanelli D., Acosta-García G., Vielle-Calzada J.P., Badillo-Corona J.A., Durán-Figueroa N. (2024). The miRNA822 loaded by ARGONAUTE9 modulates the monosporic female gametogenesis in *Arabidopsis thaliana*. Plant Reprod..

[75] Huang Y., Liu L., Chai M., Su H., Ma S., Liu K., Tian Y., Cao Z., Xi X., Zhu W. (2023). Epigenetic regulation of female germline development through ERECTA signaling pathway. New Phytol..

[76] Durán-Figueroa N., Vielle-Calzada J.P. (2010). ARGONAUTE9-dependent silencing of transposable elements in pericentromeric regions of Arabidopsis. Plant Signal Behav..

[77] Olmedo-Monfil V., Durán-Figueroa N., Arteaga-Vázquez M., Demesa-Arévalo E., Autran D., Grimanelli D., Slotkin R.K., Martienssen R.A., Vielle-Calzada J.P. (2010). Control of female gamete formation by a small RNA pathway in Arabidopsis. Nature.

[78] Peragine A., Yoshikawa M., Wu G., Albrecht H.L., Poethig R.S. (2004). SGS3 and SGS2/SDE1/RDR6 are required for juvenile development and the production of trans-acting siRNAs in Arabidopsis. Genes Dev..

[79] Yoshikawa M., Peragine A., Park M.Y., Poethig R.S. (2005). A pathway for the biogenesis of trans-acting siRNAs in Arabidopsis. Genes Dev..

[80] Himber C., Dunoyer P., Moissiard G., Ritzenthaler C., Voinnet O. (2003). Transitivity-dependent and -independent cell-to-cell movement of RNA silencing. Embo J..

[81] Hernández-Lagana E., Rodríguez-Leal D., Lúa J., Vielle-Calzada J.P. (2016). A Multigenic Network of ARGONAUTE4 Clade Members Controls Early Megaspore Formation in Arabidopsis. Genetics.

[82] Jauvion V., Elmayan T., Vaucheret H. (2010). The conserved RNA trafficking proteins HPR1 and TEX1 are involved in the production of endogenous and exogenous small interfering RNA in Arabidopsis. Plant Cell.

[83] Su Z., Zhao L., Zhao Y., Li S., Won S., Cai H., Wang L., Li Z., Chen P., Qin Y. (2017). The THO Complex Non-Cell-Autonomously Represses Female Germline Specification through the TAS3-ARF3 Module. Curr. Biol..

[84] Su Z., Wang N., Hou Z., Li B., Li D., Liu Y., Cai H., Qin Y., Chen X. (2020). Regulation of Female Germline Specification via Small RNA Mobility in Arabidopsis. Plant Cell.

[85] Komiya R., Ohyanagi H., Niihama M., Watanabe T., Nakano M., Kurata N., Nonomura K. (2014). Rice germline-specific Argonaute MEL1 protein binds to phasiRNAs generated from more than 700 lincRNAs. Plant J. Cell Mol. Biol..

[86] Nonomura K., Morohoshi A., Nakano M., Eiguchi M., Miyao A., Hirochika H., Kurata N. (2007). A germ cell specific gene of the ARGONAUTE family is essential for the progression of premeiotic mitosis and meiosis during sporogenesis in rice. Plant Cell.

[87] Singh M., Goel S., Meeley R.B., Dantec C., Parrinello H., Michaud C., Leblanc O., Grimanelli D. (2011). Production of viable gametes without meiosis in maize deficient for an ARGONAUTE protein. Plant Cell.

[88] Huettel B., Kanno T., Daxinger L., Bucher E., van der Winden J., Matzke A.J., Matzke M. (2007). RNA-directed DNA methylation mediated by DRD1 and Pol IVb: A versatile pathway for transcriptional gene silencing in plants. Biochim. Biophys. Acta.

[89] Garcia-Aguilar M., Michaud C., Leblanc O., Grimanelli D. (2010). Inactivation of a DNA methylation pathway in maize reproductive organs results in apomixis-like phenotypes. Plant Cell.

[90] Drews G.N., Lee D., Christensen C.A. (1998). Genetic analysis of female gametophyte development and function. Plant Cell.

[91] Grossniklaus U., Schneitz K. (1998). The molecular and genetic basis of ovule and megagametophyte development. Semin. Cell Dev. Biol..

[92] Christensen C.A., Subramanian S., Drews G.N. (1998). Identification of gametophytic mutations affecting female gametophyte development in Arabidopsis. Dev. Biol..

[93] Wu M.F., Tian Q., Reed J.W. (2006). Arabidopsis microRNA167 controls patterns of ARF6 and ARF8 expression, and regulates both female and male reproduction. Development.

[94] Yao X., Chen J., Zhou J., Yu H., Ge C., Zhang M., Gao X., Dai X., Yang Z.N., Zhao Y. (2019). An Essential Role for miRNA167 in Maternal Control of Embryonic and Seed Development. Plant Physiol..

[95] Xing S., Salinas M., Garcia-Molina A., Höhmann S., Berndtgen R., Huijser P. (2013). SPL8 and miR156-targeted SPL genes redundantly regulate Arabidopsis gynoecium differential patterning. Plant J. Cell Mol. Biol..

[96] Hashimoto K., Miyashima S., Sato-Nara K., Yamada T., Nakajima K. (2018). Functionally Diversified Members of the MIR165/6 Gene Family Regulate Ovule Morphogenesis in *Arabidopsis thaliana*. Plant Cell Physiol..

[97] Tucker M.R., Okada T., Hu Y., Scholefield A., Taylor J.M., Koltunow A.M. (2012). Somatic small RNA pathways promote the mitotic events of megagametogenesis during female reproductive development in Arabidopsis. Development.

[98] Cai H., Liu L., Zhang M., Chai M., Huang Y., Chen F., Yan M., Su Z., Henderson I., Palanivelu R. (2021). Spatiotemporal control of miR398 biogenesis, via chromatin remodeling and kinase signaling, ensures proper ovule development. Plant Cell.

[99] Liew L.C., Wang Y., Peirats-Llobet M., Berkowitz O., Whelan J., Lewsey M.G. (2020). Laser-Capture Microdissection RNA-Sequencing for Spatial and Temporal Tissue-Specific Gene Expression Analysis in Plants. J. Vis. Exp. JoVE.

[100] Shi D., Jouannet V., Agustí J., Kaul V., Levitsky V., Sanchez P., Mironova V.V., Greb T. (2021). Tissue-specific transcriptome profiling of the Arabidopsis inflorescence stem reveals local cellular signatures. Plant Cell.

[101] Qiao J., Jiang H., Lin Y., Shang L., Wang M., Li D., Fu X., Geisler M., Qi Y., Gao Z. (2021). A novel miR167a-OsARF6-OsAUX3 module regulates grain length and weight in rice. Mol. Plant.

[102] Chen Z., Li Y., Li P., Huang X., Chen M., Wu J., Wang L., Liu X., Li Y. (2021). MircroRNA Profiles of Early Rice Inflorescence Revealed a Specific miRNA5506 Regulating Development of Floral Organs and Female Megagametophyte in Rice. Int. J. Mol. Sci..

[103] Liu L., Chen X. (2018). Intercellular and systemic trafficking of RNAs in plants. Nat. Plants.

[104] Gandikota M., Birkenbihl R.P., Höhmann S., Cardon G.H., Saedler H., Huijser P. (2007). The miRNA156/157 recognition element in the 3’ UTR of the Arabidopsis SBP box gene SPL3 prevents early flowering by translational inhibition in seedlings. Plant J. Cell Mol. Biol..

