# Supplementary material for: Advances in Small RNA Regulation of Female Gametophyte Development in Flowering Plants
Source: Plants (Basel). 2025 Apr 23;14(9):1286. doi: 10.3390/plants14091286 (PMC12073561; doi:10.3390/plants14091286)
Supplement: Supplementary file 1 [file plants-14-01286-s001.zip › plants-3570247-supplementary.pdf]

Table S1. Classifications and functions of small RNAs in female gametophyte development

Small RNA	Target	Function
miR160	*ARF17*	*miR160*-mediated *ARF17* regulation restricts MMC formation to a single cell in *Arabidopsis* ovules[73].
miR390	*TAS3*	*miR390* mediates the regulation of MMC formation[84].
TAS3 ta-siRNA	*ARF3*	*TAS3 ta-siRNA* silences ARF3 transcription in L1 cells to control MMC specification[83].
AGO9-miR822	*At5g02350*<br>*At5g02330*<br>*At2g13900*	*AGO9-miR822* restricts megaspore survival to one cell during *Arabidopsis* megasporogenesis[74].
miR398	*AGL51/52/78*	*miR398*-mediated regulation of *AGL51/52/78* in the embryo sac to coordinate female gametophyte development and sporophyte integument formation[98].
miR165/miR166	*PHB*	*miR165/166* orchestrate ovule development by modulating *PHB* expression[96].
miR167	*ARF6*	*miR167* ensures embryo sac enclosure via *ARF6*-dependent integument development[94].
miR156	*SPL8*	*miR156*-mediated *SPL8* regulation controls female gametophyte development in *Arabidopsis*[95].

Table S2. Key mutants and phenotypes in small RNA-Mediated female gametophyte development

Small RNA mutant	Phenotype
mir160a	*miR160a* mutants exhibit high ovules abortion and supernumerary MMC-like cells[73].
mir390	*mir390* mutants produce supernumerary MMCs that retain MMC molecular characteristics[84].
TAS3 ta-siRNA insensitive transgenic mutants	*TAS3 ta-siRNA* insensitive transgenic mutants result in the production of multiple MMCs in the ovules[83].
mir822	In *mir822* mutants, impaired megaspore degradation resulted in ectopic FM formation[74].
mir398b +/- *mir398c +/-*	*miR398b*/*miR398c* double heterozygotes show ovule arrest at FG1 stage, blocking embryo sac maturation[98].
mir165/mir166	*mir165/166* mutations disrupt outer integument elongation, resulting in failure of embryo sac enclosure[96].
mir167	In *mir167* mutants, embryo sacs protruded from the micropyle and arrested development, preventing female gametophyte formation[94].
